# Robotic First Rib Resection With Adjuvant Endovascular Therapy for Chronic Paget-Schroetter Syndrome

**DOI:** 10.1016/j.atssr.2024.07.019

**Published:** 2024-07-31

**Authors:** Alejandro Zulbaran-Rojas, Miguel Montero-Baker, Nihanth Palivela, Catherine Park, Bijan Najafi, Jayer Chung, Joseph L. Mills, Bryan M. Burt

**Affiliations:** 1 Division of Vascular Surgery and Endovascular Therapy, Michael E. DeBakey Department of Surgery, Baylor College of Medicine, Houston, Texas; 2 HOPE Vascular & Podiatry, Houston, Texas; 3 Division of General Thoracic Surgery, Michael E. DeBakey Department of Surgery, Baylor College of Medicine, Houston, Texas; 4 Division of Thoracic Surgery, University of California Los Angeles, Los Angeles, California

## Abstract

**Background:**

Patients with acute Paget-Schroetter syndrome (PSS) are treated with endovascular therapy and first rib resection (FRR); however the care of patients with chronic PSS is less well understood. This report describes an emerging approach of robotic-FRR, with adjuvant endovascular therapy, for chronic PSS.

**Methods:**

A single-center, retrospective analysis was conducted of patients undergoing robotic-FRR for chronic PSS between 2017 and 2020. Chronic PSS was defined by subclavian vein (SCV) fibrosis identified on duplex ultrasound examination. Patency and clinical outcomes were compared before and after robotic-FRR.

**Results:**

Fifteen robotic-FRRs in 14 patients with chronic PSS were analyzed. Median time between acute thrombosis and presentation to our clinic was 167 days. Eleven SCVs were previously treated with anticoagulants only, and 4 had a history of thrombolysis. At the time of presentation, all SCVs displayed chronic thrombosis on duplex ultrasound; 7 (46.7%) were treated with angioplasty followed by robotic-FRR, and 8 (53.3%) proceeded first to robotic-FRR. Postoperative duplex ultrasound examination at a median of 34 days from robotic-FRR demonstrated patency in 5 (33.3%) SCVs. Postoperative angioplasty was performed in 9 (60%) SCVs after a median of 59 days from robotic-FRR, resulting in 6 (40%) additional patent SCVs. The total number of SCVs achieving patency was 11 (73.3%). There were no complications during angioplasties or robotic-FRR. Eleven patients (73.3%) achieved complete symptom resolution with decrease in swelling (*P* < .001) and pain (*P* = .016).

**Conclusions:**

Robotic-FRR in combination with adjuvant endovascular therapy was associated with favorable clinical and patency outcomes for chronic PSS.

## Visual Abstract

**Figure:**
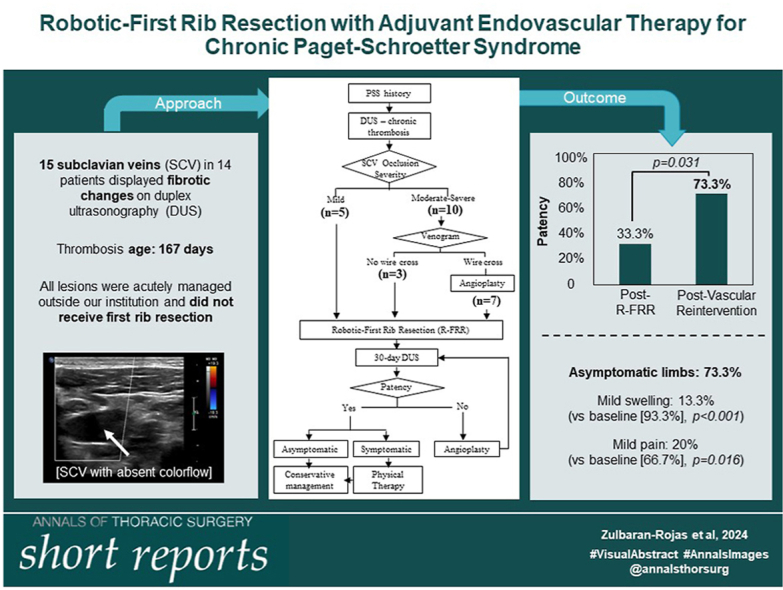

**In Short**

▪ Robotic first rib resection with adjuvant endovascular therapy for the management of chronic Paget-Schroetter syndrome resulted in 73.3% of patients achieving subclavian vein patency and symptom resolution (median of 146 days).
▪ Duplex ultrasound was useful for interrogating the severity of lesions in patients with chronic Paget-Schroetter syndrome (median thrombosis age of 167 days) and in treatment decision-making.

The treatment of patients with Paget-Schroetter syndrome (PSS) includes endovascular therapy (EVT) followed by surgical decompression with first rib resection (FRR) and systemic anticoagulation. EVT serves as the intrinsic modality to restore subclavian vein (SCV) patency through catheter-directed thrombolysis.^1^ FRR functions to remove the compressive elements on the SCV at the costoclavicular junction. Traditional surgical decompression is performed by open-FRR, which has demonstrated predictable long-term cure rates.^1^ However, the robotic transthoracic approach to FRR (robotic-FRR) gained popularity in recent years for its outstanding exposure of the first rib, elimination of retraction of the neurovascular structures, and decreased postoperative pain.^2^

Whereas the approach for acute PSS has been established, the care of patients with chronic PSS is less well understood. Controversy exists about determining chronic PSS as there is no defined time frame for the development of fibrosis. Some providers consider lesions older than 2 to 4 weeks to be chronic,^3^ and shortening the interval between catheter-directed thrombolysis and FRR may lower the risk for development of chronicity.^1^ However, others recommend waiting 1 to 3 months between these procedures to allow endothelial healing.^4^ In addition, FRR is often referred to a secondary/specialized center, which can lead to prolonged delays and subsequently chronicity of lesions, vein fibrosis, and rethrombosis.

We report the results of a care pathway that includes robotic-FRR and EVT.

## Patients and Methods

We included all patients who underwent robotic-FRR for chronic PSS from November 2017 to August 2020. This study was conducted after institutional review board approval (H-36302) at our center, waiving individual consent. Clinical characteristics, PSS treatment history outside our institution, and time of acute thrombosis to the date of presentation at our clinic were collected along with vascular interventions, complications, and period of follow-up.

We evaluated SCV patency through an upper extremity duplex ultrasound (DUS) examination performed by a registered vascular technologist on the day of initial consultation at our clinic or by review of the most recent scans obtained from external institutions (pre–robotic-FRR DUS) with interpretation by a vascular surgeon. B-mode and color Doppler were used for SCV wall evaluation and flow characteristics. Noncompressible SCVs displaying bright echogenic material filling the lumen, leading to reduced color flow and pulsed wave Doppler signals, were indicative of fibrosis/chronic thrombosis. Mild occlusions due to chronic thrombosis were those displaying reduced color flow and pulsed wave Doppler signals in a short SCV segment, with remaining flow in all other segments. Moderate occlusions were those displaying reduced color flow and blunted, pulsatile, or continuous pulsed wave Doppler signals in all SCV segments. Severe occlusions were those displaying absent color flow and pulsed wave Doppler signals in all SCV segments. Collateral network development was also collected.

After robotic-FRR, DUS examination was conducted to reassess SCV patency, defined as absence of any echogenic material that impedes blood flow velocities, with normal compressibility and pulsed wave Doppler signals. Residual lesions were described in an identical manner to the pre–robotic-FRR DUS assessment. If a secondary intervention was required to restore SCV patency, an additional DUS examination was performed for SCV reassessment.

The presence or absence of pain, numbness, and swelling in the ipsilateral upper extremity were collected from the last follow-up visit note.

### Treatment of Chronic PSS

**Figure 1:**
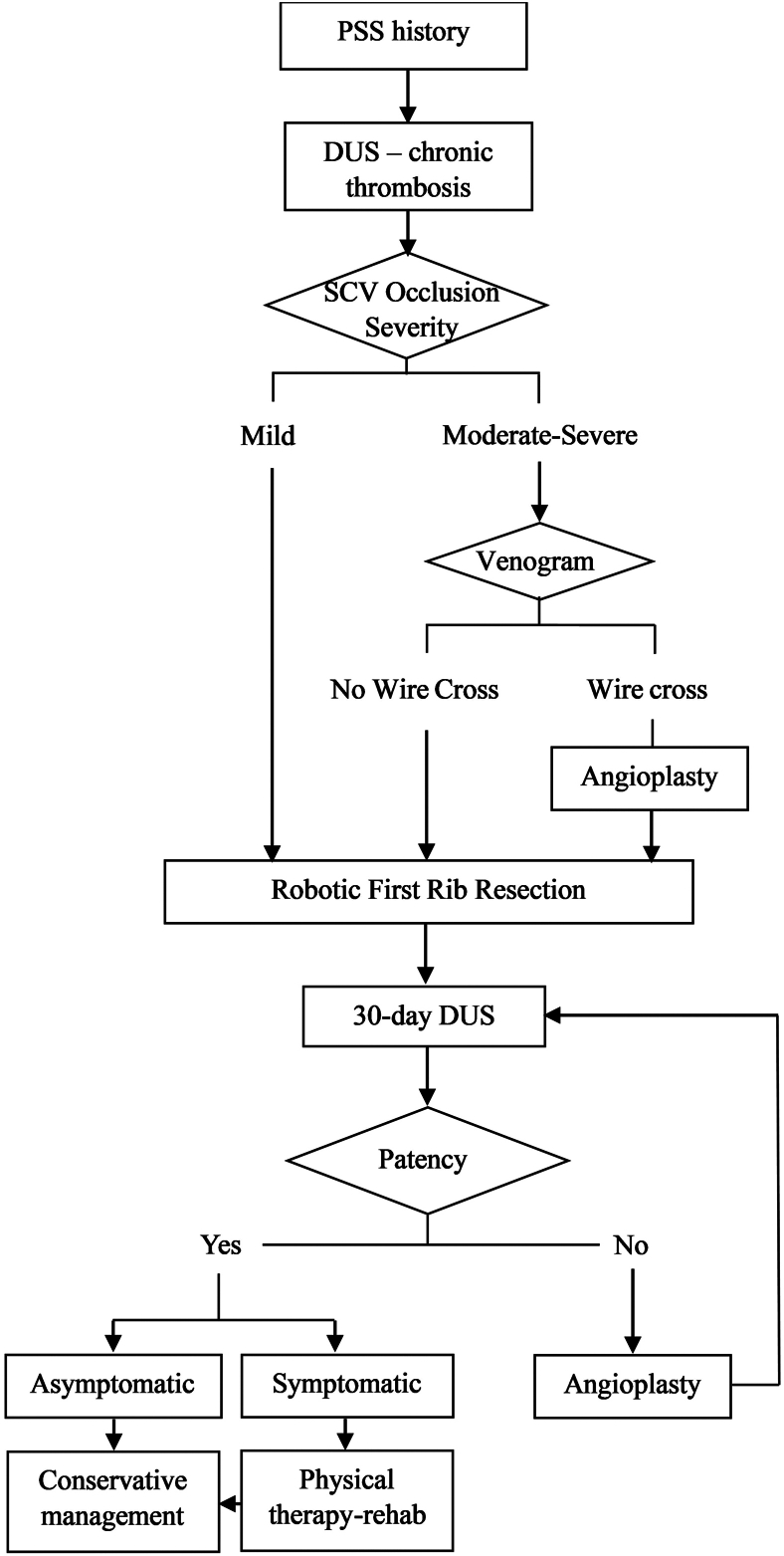
Approach to chronic Paget-Schroetter syndrome (PSS) in our institution. (DUS, duplex ultrasound; SCV, subclavian vein.)

Confirmation of chronic thrombosis is determined by the patient’s history and DUS findings (Figure 1). For SCVs displaying moderate-severe occlusion, we perform a venography. If a guidewire can cross the occluded segment, intraluminal balloon angioplasty is performed to dilate the SCV lumen. Robotic-FRR is then performed by our described technique.^2^ For SCVs displaying mild occlusion, we do not perform venography/angioplasty, and robotic-FRR is considered. After robotic-FRR, patients with significant symptoms and persisting SCV thrombosis may undergo vascular reinterventions to restore SCV patency.

### Statistical Analysis

Categorical variables were expressed as count (percentage). Continuous variables were reported as mean ± SD if they were normally distributed and median (interquartile range) if they were nonnormally distributed. Frequency of SCV patency and clinical outcomes were reported with descriptive analysis and compared by McNemar test. All tests were 2 sided, with a *P* value < .05 considered significant. The analysis was done with IBM SPSS Statistics.

## Results

Fifteen thrombotic lesions in 14 patients (aged 28 ± 11 years; 71.4% male) were evaluated. All thrombotic lesions were acutely managed outside our institution. Specifically, 11 of 15 (73.3%) were treated solely with anticoagulation, and 4 of 15 (26.7%) underwent treatment with catheter-directed thrombolysis. None of these thrombotic lesions received surgical decompression. The median duration between acute thrombosis and presentation to our clinic was 167 (122-1138) days. Of 15 limbs, 14 (93.3%) exhibited chronic swelling, 10 (66.7%) chronic pain, and 6 (40%) chronic numbness.

On baseline DUS examination (median of 30 [7-75] days before robotic-FRR), all SCVs displayed chronic thrombosis, and 14 of 15 (93.3%) had collateral network. Of 15 SCVs, 10 (66.7%) displayed severe occlusion, prompting venography. From these 10 SCVs, 3 of 10 (30%) were unsuccessfully crossed and were scheduled for elective robotic-FRR; however, 7 of 10 (70%) had successful wire crossing and received balloon angioplasty followed by robotic-FRR in a median of 41 (12-153) days. Conversely, 5 of 15 (33.3%) SCVs displayed mild occlusion and thus were initially approached by robotic-FRR. There were no complications associated with angioplasty or robotic-FRR. The length of stay after robotic-FRR was 1.6 ± 0.5 days.

### SCV Primary and Secondary Patency

Post–robotic-FRR DUS examinations were conducted at a median of 34 (25-49) days. These scans showed patency in 5 of 15 (33.3%) SCVs. However, persistent thrombosis remained in 10 of 15 (66.7%) SCVs, of which 9 of 10 (90%) still caused severe symptoms. Patients with patent SCVs or who were asymptomatic were managed without further DUS. Patients with persistent thrombosis and severe symptoms underwent a vascular reintervention, specifically balloon angioplasty (n = 8) and stent angioplasty (n = 1), at a median time of 59 (45-201) days after robotic-FRR. Reasons were attributed to partial reocclusions in 7 of 9 (77.8%) and total reocclusions noted at initial venography in 2 of 9 (22.2%); however, completion venography after angioplasty showed >90% technical success in all procedures.

Subsequently, an additional DUS examination was performed to reassess SCV patency in this subgroup at 294 (81-608) days after robotic-FRR. The results of these scans showed patency in 6 of 9 (66.7%) SCVs and persistent thrombosis in 3 of 9 (33.3%) SCVs, but with mild symptoms. The total number of SCVs achieving patency was 11 of 15 (73.3%). The comparison of SCV patency before vascular reintervention vs the cumulative SCV patency after vascular reintervention revealed a significant improvement (33.3% vs 73.3%; *P* = .031).

### Clinical Outcomes

The median follow-up from robotic-FRR to last office visit was 146 (61-804) days. At this time, 11 of 15 (73.3%) limbs were asymptomatic, whereas 4 of 15 (26.7%) patients reported mild symptoms that did not interfere with the quality of life. This outcome was accompanied by a significant reduction of limbs initially presenting with swelling (14/15 [93.3%] vs 2/15 [13.3%]; *P* < .001) and pain (10/15 [66.7%] vs 3/15 [20%]; *P* = .016).

## Comment

This study reported SCV patency and clinical outcomes of combining robotic-FRR with adjuvant EVT for the management of chronic PSS in 15 consecutive cases. This approach resulted in 73.3% of patients achieving SCV patency and complete symptom resolution evaluated at the last follow-up visit. The remaining patients had partial symptom improvement.

Endoluminal treatment and anticoagulation alone have been shown to result in unfavorable patency and symptoms in patients with PSS.^4^ Partially treated lesions can develop persistent vein wall inflammation and fibrosis, leading to chronic arm swelling, pain, or numbness, commonly known as postthrombotic syndrome.^5^ In this study, all patients presented to our institution with postthrombotic syndrome. Interestingly, none had history of surgical decompression, but 73.3% had previous treatment consisting of anticoagulants only, and 36.4% had a history of catheter-directed thrombolysis. The distortion of the native anatomy in chronic PSS results in significant reduced venous outflow capacity.^5^ This was reflected in the 93.3% of patients in our cohort who presented with chronic arm swelling and development of collateral networks of alternative venous drainage. There is currently no consensus on grading severity in postthrombotic syndrome, and the persistence of symptoms often drives these patients to seek additional care.

The election of EVT before surgical decompression should be carefully considered for chronic PSS. We used DUS to identify whether the severity of SCV chronic occlusion would allow possible EVT before robotic-FRR. This approach may identify chronic lesions that have not been totally obliterated by scarring and with greatest potential for achieving patency after FRR. During the end stage of fibrosis, the entire vein transforms into a fibrous cord with no lumen,^3^ making the clinical decision more complex.^6^ A systematic review on chronic PSS combined multiple treatment algorithms based on symptom severity.^7^ For mild symptoms accompanied by nonocclusive lesions, FRR followed by EVT was recommended. However, for severe symptoms, open vascular reconstruction was recommended.^7^ Our care pathway places emphasis on the severity of SCV patency as all patients in this series presented to our clinic with significant unresolved chronic symptoms. Of note, DUS has shown ∼21% inaccuracy, highlighting the importance of multidisciplinary assessment and clinical interpretation.^8^

**Figure 2:**
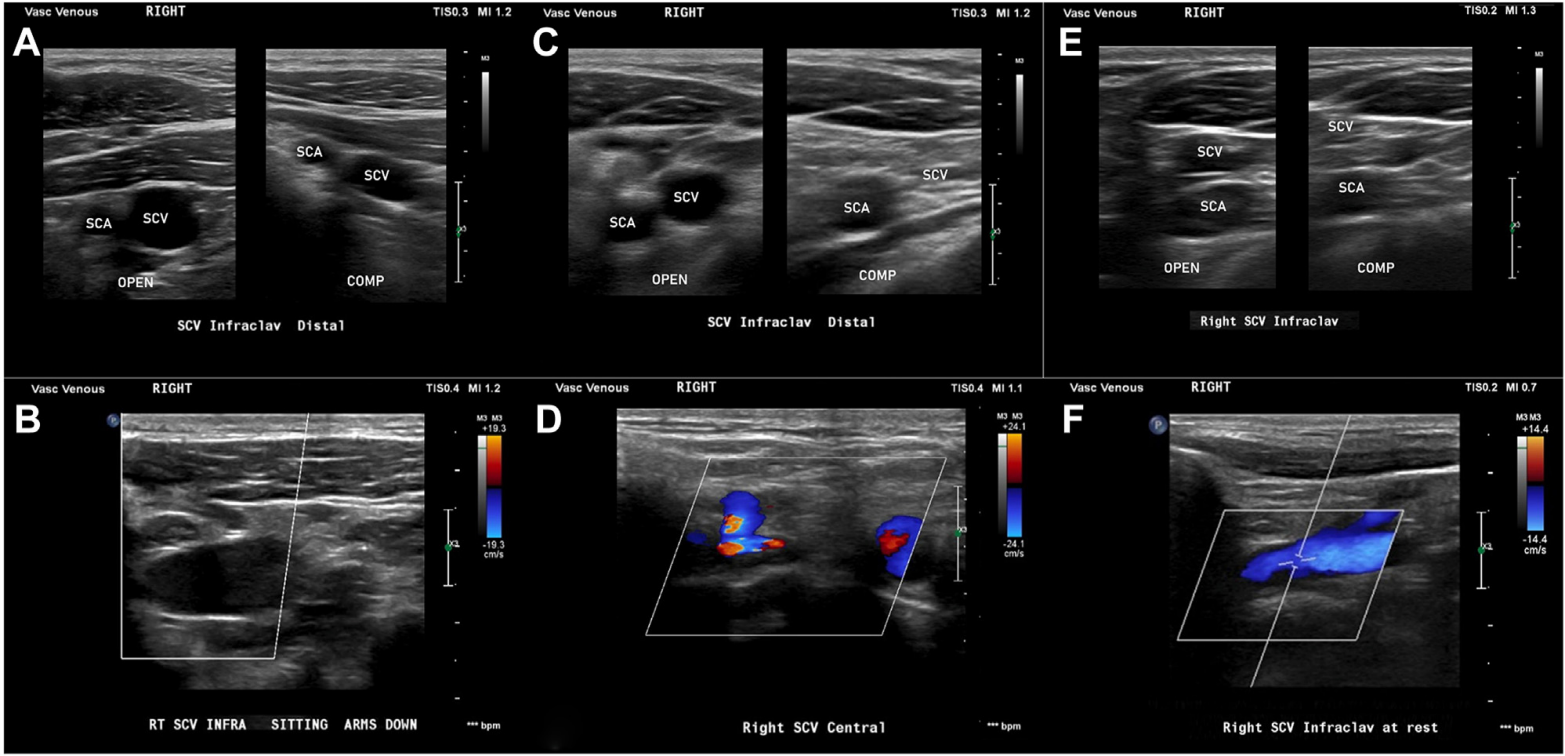
Comparison of ultrasound scans in a typical case. Before robotic first rib resection: (A) noncompressible subclavian vein (SCV) with (B) complete cessation of flow. After robotic first rib resection: (C) compressible SCV with (D) persistent stenosis in 1 SCV segment. After angioplasty: (E) compressible SCV with (F) patent SCV. (COMP, compressed; SCA, subclavian artery.)

Robotic-FRR has been shown to increase patency rates in acute and subacute PSS management,^9^ but the result of robotic-FRR for chronic PSS is not well known. Urschel and Razzuk^1^ reported that in this subpopulation, 33% of lesions remained partially occluded after open surgical decompression. In our cohort, 66.7% of the patients remained with filling defects observed at the post–robotic-FRR DUS evaluation. Therefore, a vascular reintervention was elected to achieve patency in those clinically meaningful recurrent thrombotic lesions (Figure 2). This led to a significant increase from 33% to 73.3% of SCV patency in comparing pre–vascular reintervention vs cumulative post–vascular reintervention DUS scans. In addition, 73.3% of the patients were asymptomatic at the last follow-up visit. Although these findings align with several studies addressing chronic PSS recurrent thrombotic lesions with secondary angioplasty,^6^^,^^10^ this study’s sample size was not sufficient for a confirmatory association analysis between vascular reinterventions and clinical improvement to be performed.

Recognized limitations of this study are its retrospective nature, small sample size, and lack of strict DUS surveillance, oral anticoagulant data, postoperative catheter-directed thrombolysis data, and symptom severity/postthrombotic syndrome assessment without validated surveys.

This study reported the use of DUS to identify chronic PSS and further initiate its approach by a combination of robotic-FRR with adjunctive EVT. Such a combination appeared favorable for midterm clinical and SCV patency outcomes in 15 patients. Secondary SCV angioplasty can be used to address residual lesions, to improve vein patency, and to achieve symptom resolution.

## References

[1] Urschel HC Jr, Razzuk M.A. (2000). Paget-Schroetter syndrome: what is the best management?. Ann Thorac Surg.

[2] Burt B.M., Palivela N., Cekmecelioglu D. (2021). Safety of robotic first rib resection for thoracic outlet syndrome. J Thorac Cardiovasc Surg.

[3] Molina J.E., Hunter D.W., Dietz C.A. (2009). Protocols for Paget-Schroetter syndrome and late treatment of chronic subclavian vein obstruction. Ann Thorac Surg.

[4] Habibollahi P., Zhang D., Kolber M.K., Pillai A.K. (2021). Venous thoracic outlet syndrome. Cardiovasc Diagn Ther.

[5] Persson L.M., Arnhjort T., Lärfars G., Rosfors S. (2006). Hemodynamic and morphologic evaluation of sequelae of primary upper extremity deep venous thromboses treated with anticoagulation. J Vasc Surg.

[6] Azakie A.M., McElhinney F.B., Thompson R.W., Raven R.B., Messina L.M., Stoney R.J. (1998). Surgical management of subclavian-vein effort thrombosis as a result of thoracic outlet compression. J Vasc Surg.

[7] Keir G., Marshall M.B. (2017). Management strategy for patients with chronic subclavian vein thrombosis. Ann Thorac Surg.

[8] Brownie E.R., Abuirqeba A.A., Ohman J.W., Rubin B.G., Thompson R.W. (2020). False-negative upper extremity ultrasound in the initial evaluation of patients with suspected subclavian vein thrombosis due to thoracic outlet syndrome (Paget-Schroetter syndrome). J Vasc Surg Venous Lymphat Disord.

[9] Gharagozloo F., Meyer M., Tempesta B., Gruessner S. (2019). Robotic transthoracic first-rib resection for Paget-Schroetter syndrome. Eur J Cardiothorac Surg.

[10] Chang K.Z., Likes K., Demos J., Black J.H., Freischlag J.A. (2012). Routine venography following transaxillary first rib resection and scalenectomy (FRRS) for chronic subclavian vein thrombosis ensures excellent outcomes and vein patency. Vasc Endovascular Surg.

